# Unidirectional Droplet Propulsion onto Gradient Brushes without External Energy Supply

**DOI:** 10.1021/acs.langmuir.2c03381

**Published:** 2023-02-09

**Authors:** Russell Kajouri, Panagiotis E. Theodorakis, Piotr Deuar, Rachid Bennacer, Jan Židek, Sergei A. Egorov, Andrey Milchev

**Affiliations:** † Institute of Physics, Polish Academy of Sciences, Al. Lotników 32/46, 02-668 Warsaw, Poland; ‡ Université Paris-Saclay, ENS Paris-Saclay, CNRS, LMPS, 4 Av. des Sciences, 91190 Gif-sur-Yvette, France; § Central European Institute of Technology, Brno University of Technology, Purkyňova 656/123, 612 00 Brno, Czech Republic; ∥ Department of Chemistry, University of Virginia, 22901 Charlottesville, Virginia, United States; ⊥ Institut für Physik, Johannes Gutenberg Universität Mainz, 55099 Mainz, Germany; # Leibniz-Institut für Polymerforschung, Institut Theorie der Polymere, Hohe Str. 6, 01069 Dresden, Germany; ∇ Bulgarian Academy of Sciences, Institute of Physical Chemistry, 1113 Sofia, Bulgaria

## Abstract

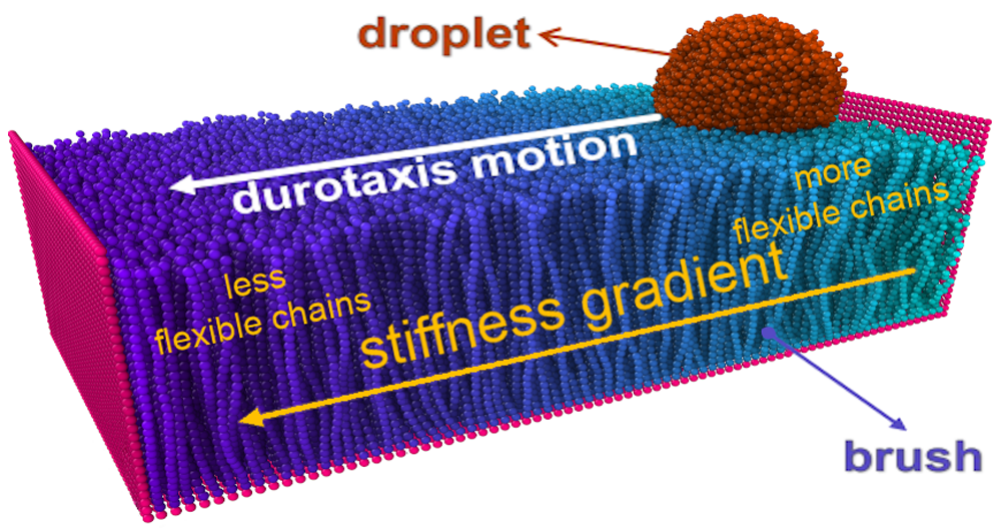

Using extensive molecular dynamics simulation of a coarse-grained model, we demonstrate the possibility of sustained unidirectional motion (durotaxis) of droplets without external energy supply when placed on a polymer brush substrate with stiffness gradient in a certain direction. The governing key parameters for the specific substrate design studied, which determine the durotaxis efficiency, are found to be the grafting density of the brush and the droplet adhesion to the brush surface, whereas the strength of the stiffness gradient, the viscosity of the droplet, or the length of the polymer chains of the brush have only a minor effect on the process. It is shown that this durotaxial motion is driven by the steady increase of the interfacial energy between droplet and brush as the droplet moves from softer to stiffer parts of the substrate whereby the mean driving force gradually declines with decreasing roughness of the brush surface. We anticipate that our findings indicate further possibilities in the area of nanoscale motion without external energy supply.

## Introduction

The motion of nano-objects, for example, liquid nanodroplets, can be provoked and sustained on solid substrates without an external energy supply. Moreover, the direction of motion can be controlled, and nanodroplets can move along predetermined trajectories. A way of achieving this effect is by placing the droplet onto a *gradient* substrate, that is, a substrate with a steadily varying property along a specific direction. This is particularly attractive for the development of various technologies in microfluidics, microfabrication, coatings, nanoscale actuation and energy conversion, and biology.^1−12^ Various possibilities for the design of gradient substrates have been reported. For example, durotaxis motion is caused by changes in stiffness along a substrate, as has been shown in various natural processes in biology (e.g., cell movement on tissues)^11,12^ and in the case of real and in silico experiments with liquid droplets.^13−23^ Another characteristic example is the rugotaxis motion of droplets on wavy substrates with a gradient in the wavelength that characterizes their pattern.^24,25^ Other possibilities include the use of wettability differences^26,27^ and physical pinning.^28^ Recent work has also highlighted the possibility of unidirectional transport of small condensate droplets on asymmetric pillars^29^ or three-dimensional capillary ratchets.^30^ In the latter case, the motion can take place in one or the other direction, depending on the surface tension of the liquid. Other possibilities of directional motion can take advantage of charge gradients that can achieve long-range transport and are based on electrostatic^31,32^ or triboelectric charges.^33^ In contrast, motion caused by temperature gradient (thermotaxis),^34^ electrical current,^35−38^ charge,^39−41^ or even simple stretch,^42^ would require external energy supply,^19^ as, also, in the case of chemically driven droplets,^43,44^ droplets on vibrated substrates,^45−48^ or wettability ratchets.^49−52^

Inspired by our previous work with specific substrate designs that lead to the durotaxis^13^ and rugotaxis^24^ motion of nanodroplets as motivated by the corresponding experiments,^15,25^ here, we propose a new design for the substrate, which is capable of sustaining the droplet motion. We consider a polymer brush, consisting of polymer chains grafted onto a flat, solid surface, and the stiffness gradient is introduced to the brush substrate by varying the stiffness of the polymer chains, which in practice amounts to tuning their persistence length. To understand the mechanism of the durotaxis motion on brush substrates and analyze the influence of relevant parameters for the brush and the droplet (e.g., droplet adhesion to the substrate, droplet size, viscosity, etc.), we have carried out extensive molecular dynamics (MD) simulations of a coarse-grained (CG) model. This is crucial, as the nanoscale motion of nano-objects is usually controlled by tiny effects at the interface between the droplet and the substrate resulting from the molecular interactions between the two, which only a method with molecular-scale resolution can capture. As in the case of durotaxis^13^ and rugotaxis^24^ motions, we find that the motion is caused by a gradient in the droplet–substrate interfacial energy, which translates into an effective force that drives the droplet toward the stiffer, flatter parts of the substrate. Moreover, we find that the efficiency of the durotaxis motion for brush substrates is higher for moderate values of the grafting density and droplet adhesion to the substrate as well as for smaller droplets and longer brush chains. Surprisingly, we have not observed a significant effect of the stiffness gradient in the case of brush substrates when the motion was successful. We anticipate that our study will shed some light into the durotaxis motion of droplets on brush, gradient substrates, thus providing further possibilities in nanoscale science and technology,^20^ for various medicine and engineering applications.^12,53^ Moreover, brush substrates share connection with various biological surfaces that expel various exogenous substances from their structure,^54^ such as the mucus layer from airway epithelia,^55^ while the gradient concept plays an important role in applications of regenerative medicine.^12^ In the following, we discuss our simulation model and methodology. Then, we will present and discuss our results, and in the final section we will draw our conclusions.

## Materials and Methods

**Figure 1:**
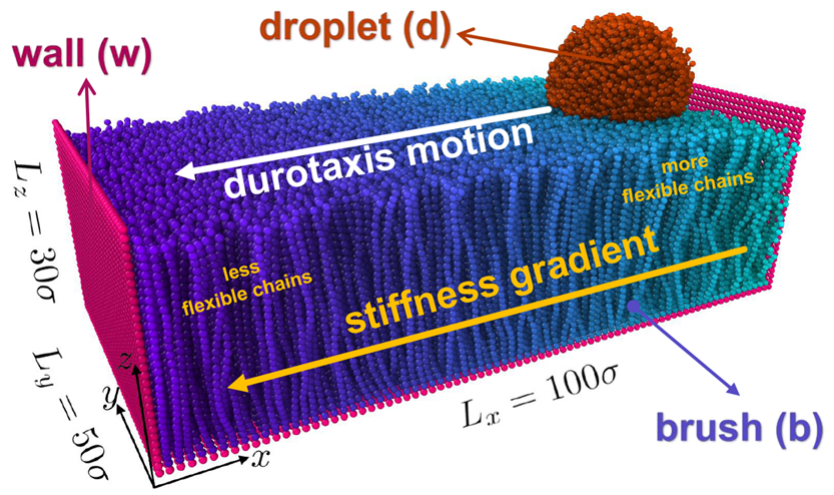
Typical initial configuration of the system, where the droplet is placed on the softest end of the brush substrate. Here, *N*_b_ = 30, *N* = 4000, and *N*_d_ = 10 beads, σ_g_ = 0.6 σ^–2^, and ε_db_ = 0.6 ϵ. At the softest end *k*_θ_ = 0 ϵ/rad^2^, while at the stiffest end *k*_θ_ = 80 ϵ/rad^2^, with a linear gradient of *k*_θ_ between them in the *x* direction. *L*_*x*_, *L*_*y*_, and *L*_*z*_ indicate the dimensions of the immobile walls. See text for further details. The snapshot of the system was obtained using Ovito software.^56^

Our system consists of a polymer-brush substrate and a droplet placed on its soft part (Figure 1). We have found that the durotaxis motion in the case of brush substrates takes place from the softer toward its stiffer parts, which is in line with previous simulation findings for another substrate design with stiffness gradient.^13^ In the direction of the stiffness gradient, the substrate has length *L*_*x*_ = 100 σ (σ is the unit of length), while in the *y*-direction, *L*_*y*_ = 50 σ, which guarantees that mirror images of the droplet in this direction will not interact during the course of the simulation due to the presence of periodic boundary conditions that are applied in all Cartesian directions. Finally, two walls are placed normal to the *x* direction as shown in Figure 1, and the size of the box in the *x* direction is large enough to guarantee that there are no interactions between the walls or the polymers on the two opposite sides of the simulation domain in the *x* direction. Wall beads were kept immobile during the simulation.

The standard bead–spring model^57^ has been employed in our simulations. In this model, interactions between any of the system components, i.e., the drop (d), the brush (b), and the wall (w) beads, are described by means of the Lennard-Jones (LJ) potential
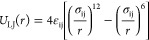
1where *r* is the distance between any pair of beads in the system within a cutoff distance. Indices i and j in eq 1 indicate the type of beads. The size of the beads is σ_ij_ = σ for all interactions. The LJ potential is cut and shifted at the cutoff distance *r*_c_ = 2.5 σ, for the interaction between the droplet (d) beads, as well as the interaction between the droplet and the brush (b) beads. In contrast, a purely repulsive potential for the interaction between the brush beads, as well as between the brush and the wall (w) beads, was considered, that is, in this case, *r*_c_ = 2^1/6^ σ. The strength of the attractive interactions is determined by the parameter ε_ij_ of the LJ potential.^58^ In our study, ε_dd_ = 1.5 ϵ, with ϵ defining the energy scale. Moreover, ε_bb_ = ϵ, while ε_db_ is the parameter that controls the attraction (adhesion) of the droplet to the substrate beads and in our study ranged from 0.1 to 1.2 ϵ.

The grafting density σ_g_ is varied from 0.1 to 1.0 σ^–2^ in our study. The size of the droplets can also vary through the total number of beads that the droplet contains, which ranged between 2 × 10^3^ and 1.6 × 10^4^ beads in our simulations. These beads belong to fully flexible, linear polymer chains. By varying the length of the droplet chains, e.g., from 10 to 80 beads, we can alter the viscosity of the droplet.^13^ The finite extensible nonlinear elastic (FENE) potential^57^ was used to tether together consecutive beads in these polymer chains as well as the polymer beads along the linear brush-polymer chains. The mathematical expression for the FENE potential is as follows
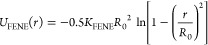
2where *r* is the distance between two consecutive beads along the polymer backbone, while *R*_0_ = 1.5 σ expresses the maximum extension of the bond, and *K*_FENE_ = 30 ϵ/σ^2^ is an elastic constant. Lengths of the polymer chains in the droplet greater than *N*_d_ = 10 guarantee that there are no evaporation effects and the vapor pressure is hence sufficiently low.^59^ We have also investigated the effect of the length *N*_b_ of the polymer chains of the brush on the durotaxis motion, by choosing different lengths, namely, *N*_b_ = 15, 30, and 50 beads.

The stiffness gradient is imposed on the brush substrate by varying the stiffness of the individual brush polymer chains. The total length of the brush chains *N*_b_ was the same for all chains, but their stiffness changed depending on the Cartesian coordinate of their grafting site in the *x* direction; i.e., chains with the same position *X* of their grafted end have the same stiffness. The chain stiffness was controlled by using a harmonic angle potential for every triad of consecutive beads along the polymer chain and tuning its strength through the harmonic constant *k*_θ_. The form of the harmonic potential reads
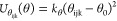
3where θ_ijk_ is the angle between three consecutive beads i, j, and k along a brush polymer chain, and θ_0_ = π rad is the equilibrium angle. A linear gradient in the stiffness constant *k*_θ_ is considered in our study to explore the properties of our systems. As we will discuss later, while the gradient in the stiffness of the substrate is necessary to initiate and maintain the durotaxis motion, the system is rather insensitive to the exact value of the gradient, and the key parameters for the brush substrate turn out to be the grafting density σ_g_ and the substrate wettability as controlled via the parameter ε_db_. The reasons for this will be revealed during the discussion of our results.

To evolve our system in time, the Langevin thermostat was used, whose details have been discussed in previous studies.^60,61^ Hence, the simulations are in practice realized in the canonical ensemble,^62^ where the temperature *T* of the system fluctuates around a predefined value *T* = ϵ/*k*_B_, with *k*_B_ being the Boltzmann constant and ϵ the energy unit. For the integration of the equations of motion, the LAMMPS package^63^ was employed. The MD time unit is , where *m* is the unit of mass, and the integration time step was Δ*t* = 0.005 τ. Typical simulation trajectories start from configurations like the one presented in Figure 1 with the total length of each trajectory being 10^8^ MD integration steps. If a droplet fully transverses the substrate from the softest to the stiffest end of the substrate, then the durotaxis motion is considered as successful. To ensure reliable statistics an ensemble of ten independent trajectories with different initial conditions (by changing the initial velocities assigned to each particle) was used for each set of system parameters. Our results are based on the analysis of these trajectories for each case.

## Results and Discussion

**Figure 2:**
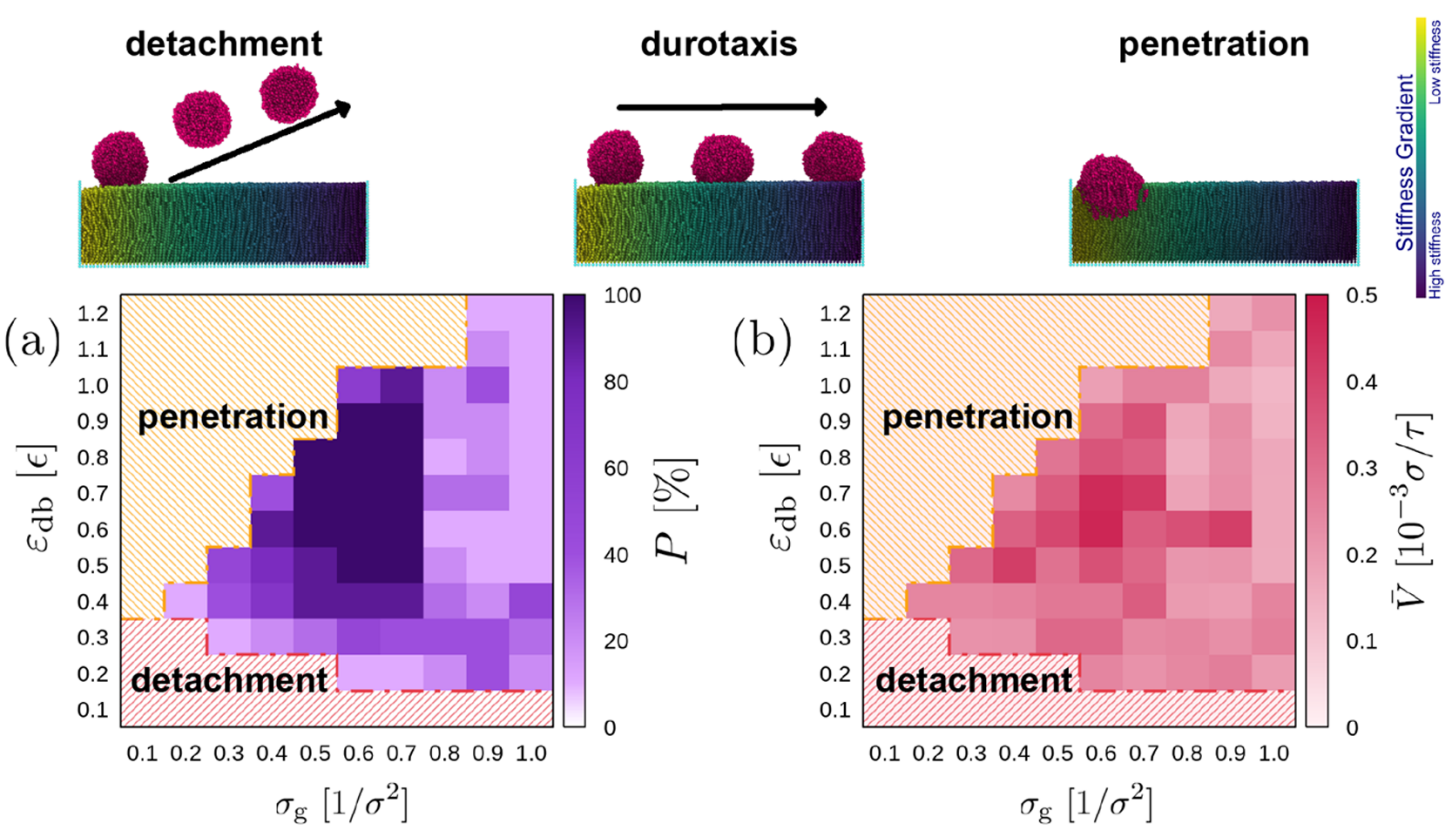
(a) Regime map indicating the probability *P* (color scale) that a droplet will cover the full distance over the substrate in the *x* direction from the softest to the stiffest part (successful durotaxis cases) for different values of the droplet–substrate attraction ε_db_ and the grafting density σ_g_. Probabilities *P* are based on an ensemble of ten independent simulations for each set of parameters. The regimes where the droplet penetrates into the brush or detaches from the substrate due to the weak ε_db_ attraction are also shown with a different color. (b) The color map indicates the average velocity of the droplet, *v̅* = *L*_*x*_^′^/*t*, for the successful durotaxis cases, where *t* is the time that the droplet needs to cross the full length of the brush substrate in the *x* direction, and *L*_*x*_^′^ is the actual distance covered by the center-of-mass of the droplet for each successful case. *N* = 4000, *N*_d_ = 10, *N*_b_ = 30 beads. The stiffness constant for the polymer chains in the softest part of the substrate is zero (fully flexible chains), growing linearly to *k*_θ_ = 80 ϵ/rad^2^ at the stiffest part of the substrate. Since *L*_*x*_ = 100 σ, the stiffness gradient is Γ = 0.8 ϵ/rad^2^σ. Snapshots on top of the plot indicate examples of detachment, durotaxis, and penetration, as indicated.

**Figure 3:**
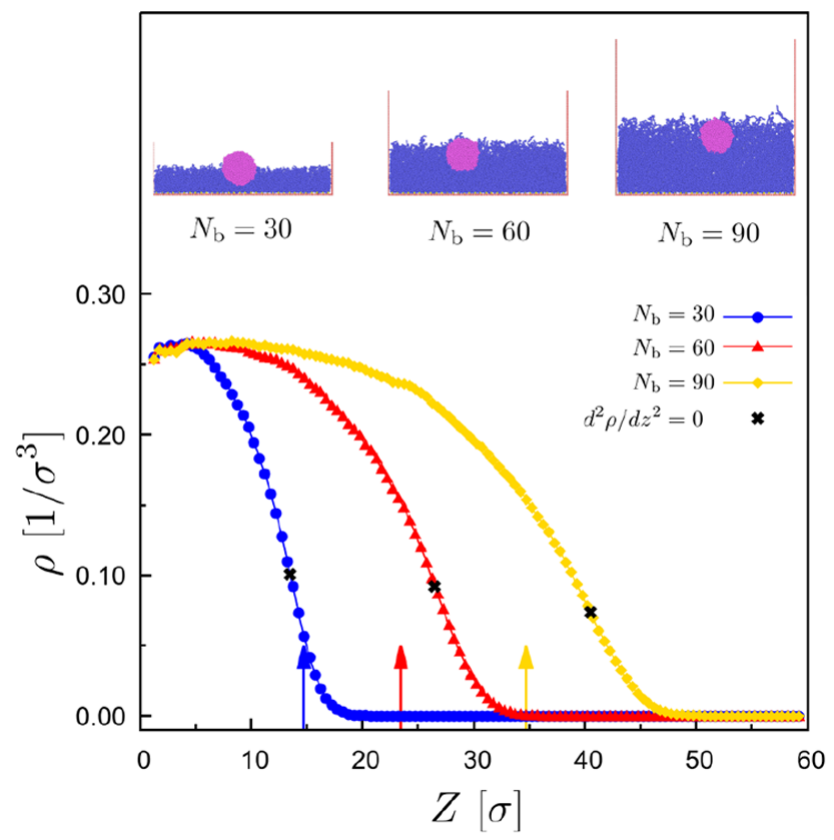
Density profile in the *z* direction for three polymer brushes at σ_g_ = 0.6 σ with fully flexible chains of length, *N*_b_ = 30, 60, and 90 beads, as indicated. The inflection point of the curves, d^2^ρ/d*z*^2^ = 0, marked with (×), is shown for each case, which corresponds to the height of the brush. The position of the center of mass of the droplet in the *z* direction is marked with arrows of the same color for each case. Here, *N* = 4000, *N*_d_ = 10 beads, and ε_db_ = 0.6 ϵ. Snapshots for each case are shown in the plot. In the case of brushes with longer chains, the droplet is immersed deeper into the brush.

By exploring a wide range of parameters, we have found that the grafting density σ_g_ and the attraction strength between the droplet and the substrate ε_db_ are two key parameters of the substrate design, since they greatly affect the possibility for successful durotaxis. Figure 2 presents the regime maps as a function of these two parameters with the probability *P* of successful durotaxis calculated from the ensemble of ten independent simulations for each set of parameters. However, in our in silico experiments, apart from durotaxis motion, we have also documented situations in which the droplet penetrates into the substrate or detaches from it. Our results indicate that small attraction strengths will lead to droplet detachment. This is more probable at smaller grafting densities due to fewer brush–droplet interactions. In contrast, large values of the attraction strength ε_db_ can lead to the penetration of the brush substrate by the droplet, again for smaller values of the grafting density σ_g_. Moreover, our results suggest that the difficulty of the droplet to penetrate into the substrate increases roughly linearly with the grafting density up to ε_db_ = ϵ, as evidenced by the linear boundary in the regime maps. In fact, penetration becomes impossible when σ_g_ ≥ 0.9 σ^–2^, since there is not enough space among the brush beads to accommodate additional droplet beads. Moreover, the degree of brush penetration also depends on the length *N*_b_ of the brush chains, as shown in Figure 3. In general, our data suggest that droplets are immersed deeper in brushes with longer chains, as shown here in the case of fully flexible polymers and judging by the center-of-mass of the droplet with respect to the position of the brush surface, as defined by the inflection point at the density profile (Figure 3). A more detailed study on this effect could potentially reveal more details for droplets immersed in brush substrates, but this clearly goes beyond the scope of our current study.

When penetration and detachment of the droplet are avoided, then persistent durotaxis motion is observed with a certain probability *P*, which depends on the choice of σ_g_ and ε_db_ (Figure 2a). From the results of Figure 2a, we find that the range 0.5 σ^–2^ ≤ σ_g_ ≤ 0.7 σ^–2^ combined with 0.5 ϵ ≤ ε_db_ ≤ 0.9 ϵ, in general, provides certainty in the success of the durotaxis motion (*P* = 100%), since all our droplets were able to fully cross the substrate from the softest to the stiffest parts of the brush within the available simulation time of 10^8^ MD time steps. As the grafting density increases above 0.7 σ^–2^, however, we observe that the probability of durotaxis success suddenly decreases. In this case, a higher density of the brush chains increases the resultant brush stiffness owing to the close packing of the chains, which leads to a situation that the role of the nominal stiffness of the individual brush chains *k*_θ_ in determining the effective stiffness gradient becomes negligible. As we will see later in our discussion concerning the underlying durotaxis mechanisms, the extent of disarray of the brush chain end-monomers at the brush surface is also reduced pointing to a rather flat density profile.

To determine the efficiency of the durotaxis motion, we have computed the average velocity *v̅* of the droplet for the successful cases for each set of parameters σ_g_ and ε_db_ (Figure 2b). Our results indicate that the probability *P* for success rather correlates with the highest average velocity *v̅*, but large values of *v̅* can also be obtained in certain cases where *P* < 100%; for example, the case σ_g_ = 0.9 σ^–2^, ε_db_ ≈ 0.6 ϵ (Figure 2b). This is a clear indication that durotaxis motion is controlled by tiny effects that can greatly influence the outcome of the experiments. Moreover, obtaining reliable statistics in cases of *P* < 100% remains a challenge in MD since this would require the realization of a large number of simulations. Hence, as *P* → 0 obtaining reliable statistics becomes more of a challenge, and outliers in the statistics are more probable. In summary, the plots of Figure 2 suggest that, if one would like all droplets to fully cross the substrate in the direction of the stiffness gradient in the shortest time, then values of σ_g_ = 0.6 σ^–2^ and ε_db_ ≈ 0.7 ϵ would constitute an optimal choice in the in silico experiments. Hence, we argue that moderate values of σ_g_ and ε_db_ favor successful and efficient (in terms of time to cross the whole substrate) durotaxis motion.

**Figure 4:**
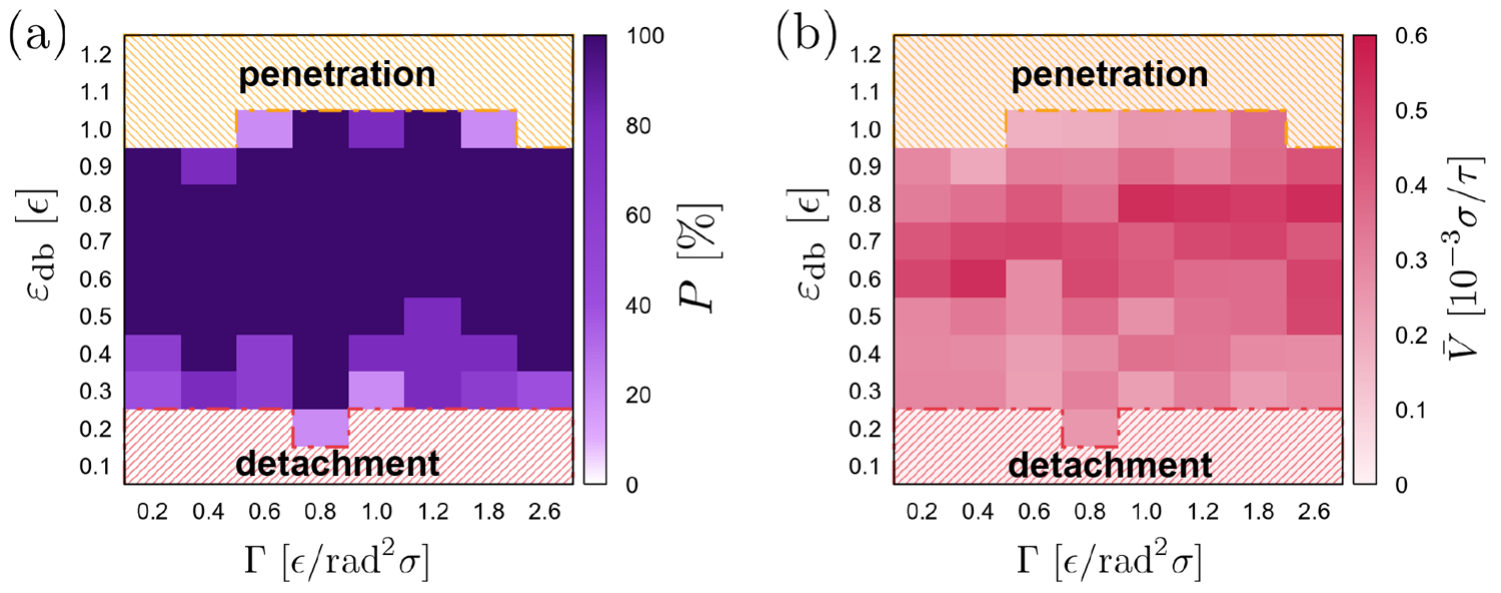
(a) Regime map indicating the probability *P* (color scale) that a droplet will cover the full distance over the substrate in the *x* direction from the softest to the stiffest part (successful durotaxis cases) for different values of the droplet–substrate attraction ε_db_ and the stiffness gradient Γ = d*k*_θ_/d*x*. The regimes where the droplet penetrates into the brush or detaches from the substrate due to the weak ε_db_ attraction are also shown with a different color. (b) The color map indicates the average velocity of the droplet *v̅* = *L*_*x*_^′^/*t* for the successful durotaxis cases, where *t* is the time that the droplet needs to cross the full length of the brush substrate in the *x* direction, and *L*_*x*_^′^ is the actual distance covered by the center-of-mass of the droplet for each successful case. The stiffness constant *k*_θ_ for the polymer chains in the softest part of the substrate is zero (fully flexible chains), growing linearly to its highest value at *x* = *L*_*x*_ = 100 σ, which depends on the chosen stiffness gradient, Γ. Here, σ_g_ = 0.6 σ^–2^, *N* = 4000, *N*_b_ = 30, and *N*_d_ = 10 beads.

Finally, we have explored the effect of the stiffness gradient on the durotaxis motion. Here, we have picked the best case of Figure 2, that is σ_g_ = 0.6 σ^–2^ and ε_db_ = 0.6 ϵ, and varied the stiffness gradient Γ = d*k*_θ_/d*x* in the range of 0.2–2.6 for fully flexible chains at the softest part of the substrate, which has actually provided the best result in terms of durotaxis success and efficiency. Overall, we have found that durotaxis is insensitive to the value of Γ in the range of 0.5 ϵ ≤ ε_db_ ≤ 0.9 ϵ (Figure 4a), in contrast to what has been observed for other in silico substrate designs.^13^ Moreover, the average velocities are spread out with small variations and no indication of a clear trend (Figure 4b) that would indicate that a larger stiffness gradient would lead to more efficient durotaxis motion exists, which has been the case for other substrate designs.^13^ Moreover, since the motion is most efficient when the softest part consists of fully flexible chains (*k*_θ_ = 0 ϵ/rad^2^) might suggest that brush substrates with polymer chains of small persistence lengths (as soft as possible) are more suitable for successful durotaxis motion, henceforth, all our results refer to brush substrates with fully flexible chains at their softest part and stiffness gradient Γ = 0.8 ϵ/rad^2^σ.

**Figure 5:**
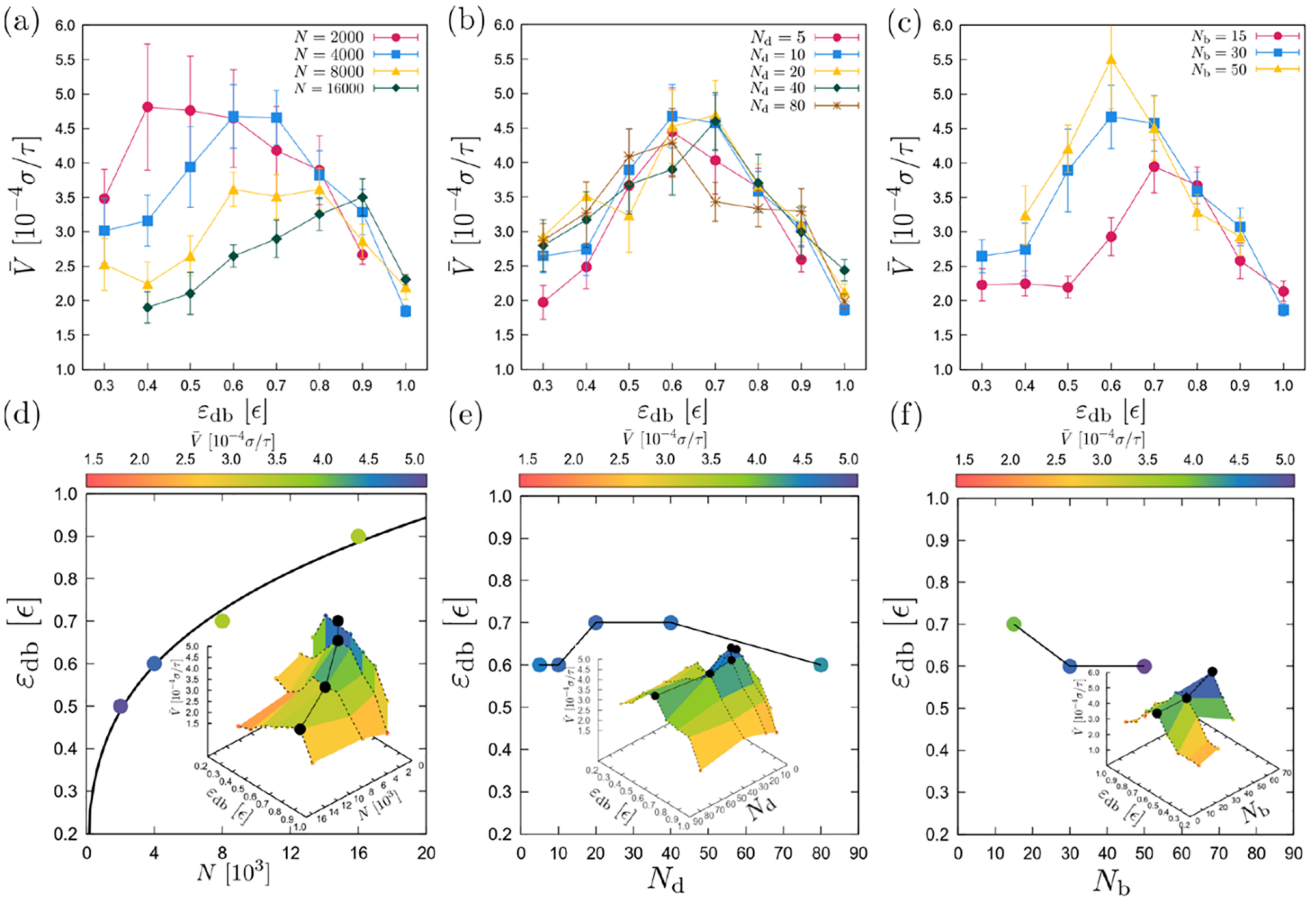
Average droplet velocity *v̅* as a function of ε_db_ for different (a) droplet size *N* (*N*_d_ = 10, *N*_b_ = 30 beads), (b) chain length of the polymer chains of the droplet *N*_d_ (*N* = 4000 and *N*_b_ = 30 beads), and (c) brush polymer chain length *N*_b_ (*N* = 4000 and *N*_d_ = 10 beads), as indicated. (d) Documented maximum average velocity *v̅*, indicated by the color map, as a function of ε_db_ and *N*. (inset) Average velocity *v̅* for all pairs of (ε_db_, *N*). The black points indicate the pairs (ε_db_, *N*) for which we have the maximum average velocity *v̅*, which is shown in the main plot. (e) Same as (d), but data are plotted as a function of (ε_db_, *N*_d_). (f) In this case data are plotted as a function of (ε_db_, *N*_b_). σ_g_ = 0.6 σ^–2^, Γ = 0.8 ϵ/rad^2^σ in all cases. (a–c) Data as a function of ε_db_ with one other quantity varying, the others held constant. Therefore, it does take care to carry out a systematic study with as much held constant between data as useful. (d–f) Visualization of the same data as in the top panels but with a different visualization in terms of maximum velocities.

In the following, we examine the effect of various parameters on the efficiency of the durotaxis motion. For our analysis, we have picked the case σ_g_ = 0.6 σ^–2^, which has shown the best performance in terms of the probability *P* and the average velocity *v̅* in our study (Figure 2) and therefore would most probably allow for exploring a wider range of the parameter space. Then, Figure 5a,d illustrates the dependence of the average velocity *v̅* on the attraction strength ε_db_ for various droplet sizes *N*. We observe that the fastest durotaxis motion takes place for the smallest droplets, namely, *N* = 2000 beads. As ε_db_ increases, a maximum value of the average velocity *v̅* appears for each case with droplets of different size. In addition, we consistently see that this maximum value of *v̅* becomes smaller for larger droplets. For example, for the droplet of *N* = 2000 beads the average velocity measured over ten trajectories is *v̅* ≈ 4.7 × 10^–4^ σ/τ, while *v̅* ≈ 3.5 × 10^–4^ σ/τ for droplets with 16 000 beads. These differences are generally considered small, especially compared to other in silico experiments.^13,24^ Moreover, we see that the maximum shifts to higher values of the attraction strength ε_db_ as the size of the droplet increases. For example, the maximum velocity is observed when ε_db_ = 0.4 ϵ for droplets with *N* = 2000 beads and for ε_db_ = 0.9 ϵ for droplets of 16 000 beads (Figure 5d). Finally, for small droplets we observe a steep increase of the average velocity with ε_db_ and then a slow decrease (Figure 3a). For medium-size droplets, (i.e., *N* = 4000 and *N* = 8000 beads), there is a smooth maximum that develops in the middle range of ε_db_, i.e., 0.6 ϵ ≤ ε_db_ ≤ 0.7 ϵ, while in the case of droplets with *N* = 16 000 beads there is a maximum that slowly develops as ε_db_ increases, which is followed by a steeper decrease when ε_db_ > 0.9 ϵ. In summary, we observe that the size of the droplets is an important parameter for the durotaxis motion.

The next parameter to examine is the chain length of the polymers comprising the droplet *N*_d_ (Figure 5b,e). In practice, longer chain lengths would result in a larger droplet viscosity. During this and previous work with polymer liquid droplets^13^ we have determined that the most relevant values for our study are within the range of 10 ≤ *N*_d_ ≤ 80 beads. Interestingly, the droplet viscosity seems not to play an important role in the overall efficiency of the durotaxis motion, for a given attraction strength ε_db_. In other words, droplets with different *N*_d_ would exhibit a similar durotaxial efficiency for a specific choice of ε_db_. As a result, the maximum average velocity *v̅* as a function of ε_db_ appears at ε_db_ ≈ 0.6–0.7 ϵ (Figure 5e). Hence, we can conclude that droplets with different viscosity will have a similar durotaxis performance and, here, a moderate choice for the value of droplet–substrate attraction strength would yield the fastest durotaxis motion.

The effect of the length of the brush chains *N*_b_ on the durotaxis motion is shown in Figure 5c,f. We find that the larger the length *N*_b_, the more efficient the durotaxis motion becomes. Although a larger difference is noticed when *N*_b_ was doubled from 15 to 30 beads, a saturation in our data occurred when *N*_b_ increased from 30 to 50 beads. Overall, our results indicate that brushes with longer polymer chains favor the durotaxis motion. Moreover, for *N*_b_ = 30 and 50 beads, the maximum velocity is found for ε_db_ = 0.6 ϵ and, hence, is independent of the choice of *N*_b_. The reasons for this behavior will become more apparent as we discuss the underlying mechanism of the droplet motion in the following.

**Figure 6:**
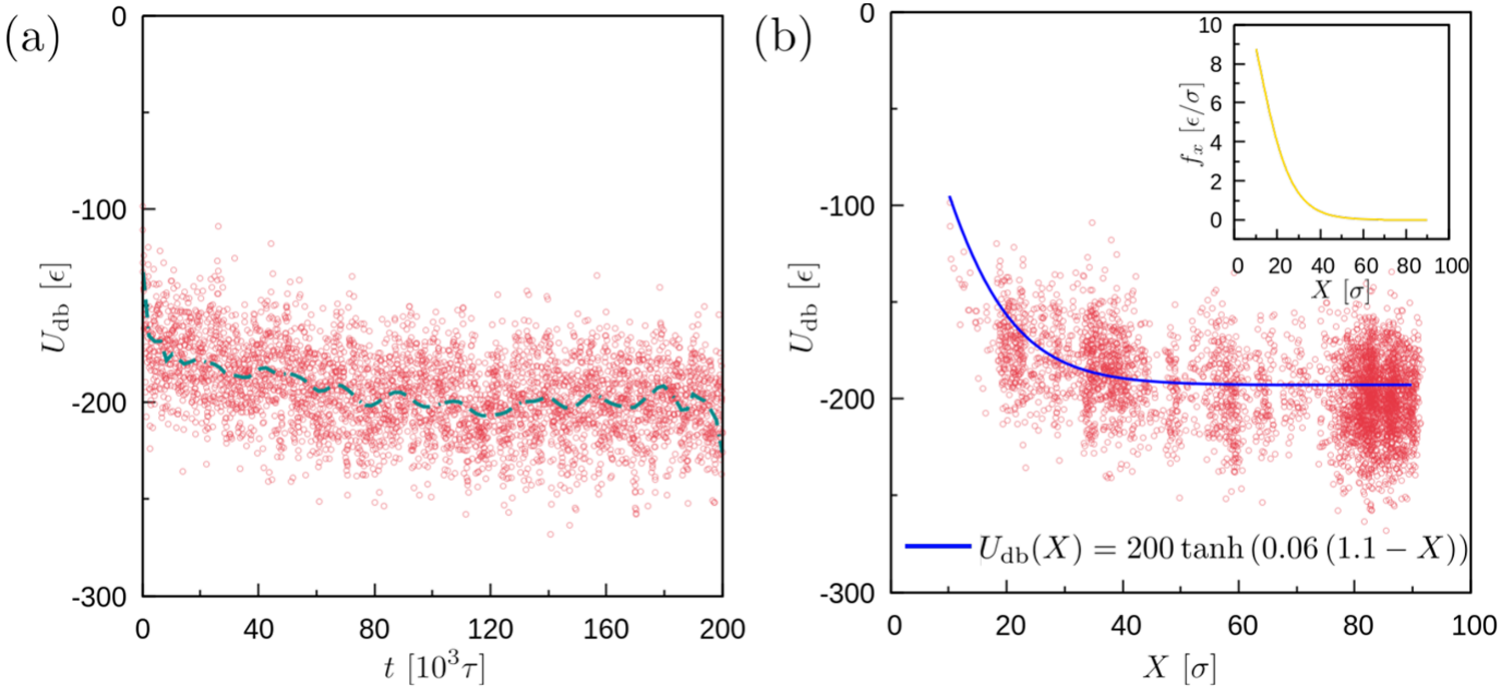
(a) Droplet–Brush interfacial energy *U*_db_ as a function of time, *t* (dashed line is a guide for the eye), and (b) the position *X* of the center of mass of the droplet in the *x* direction, for a case with successful durotaxis (*N* = 4000, *N*_d_ = 10, and *N*_d_ = 30 beads. ε_db_ = 0.6 ϵ and σ_g_ = 0.6 σ^–2^). (inset) The force  based on a nonlinear fit of the tanh function on the *U*_db_ data. The higher concentration of points in panel (b) at certain ranges of *X* simply indicates that the droplet spends more time at these positions as it moves to the stiffest parts of the substrate. The fit function of the *U*_db_ only provides an average picture of the decay of the interfacial energy.

From our earlier studies, we have seen that the minimization of the interfacial energy *U*_db_ between the droplet and the substrate is the driving force for the durotaxis motion in the case of substrates with stiffness gradient^13^ or wrinkled substrates with a gradient in the wavelength characterizing the wrinkles.^24^ This has also been found in the case of a nanoflake on substrates with stiffness gradient.^16^ Moreover, we have argued that the efficiency of the motion depends on the rate of change of the interfacial energy along the gradient direction. Hence, it is relevant to examine the interfacial energy *U*_db_ as a function of time *t* and the coordinate *X* of the center-of-mass of the droplet along the substrate (see Figure 6 showing these quantities for a successful durotaxis case). Our results indicate that *U*_db_ over time reaches more negative values (Figure 6a), which corresponds to a larger number of attractive pair interactions between the droplet and the substrate. Importantly, we also see that the energy decreases as a function of the position of the droplet *X*, which clearly indicates that the droplet moves to areas of more negative energy (Figure 6b) toward the stiffer parts of the substrate (increasing *X*). A larger decrease of the energy takes place in the initial soft parts of the substrate at small *X* values, while the change in the substrate–droplet interfacial energy is smaller after the droplet has moved a distance of about 40 σ. Moreover, we can see that the droplet does not spend the same time at each position *X*, as seen by the density of points at certain positions *X* and knowing that samples have been taken over time at equal intervals. Hence, the durotaxis motion of the droplet cannot be characterized as a steady state. To illustrate the effect of the gradient in the interfacial energy *U*_db_, one can actually plot the negative derivative of the interfacial energy (inset of Figure 6b) after performing a suitable fit on the *U*_db_. This derivative would correspond to an on-average driving force  that propels the droplet toward the stiffer parts of the brush substrate. It is clear that this “force” becomes significantly smaller when the droplet enters the stiffer half of the substrate in the *x* direction. However, this small force is still able to sustain the motion of the droplet and lead to a successful durotaxis case. Still, the inset of Figure 6b reflects the observed behavior caused by an average force that propels the droplet. Finally, we have clearly seen from our data that unsuccessful durotaxis cases are characterized by a flat interfacial energy that fluctuates around a constant value, which would ideally yield *f*_*x*_ ≈ 0.

**Figure 7:**
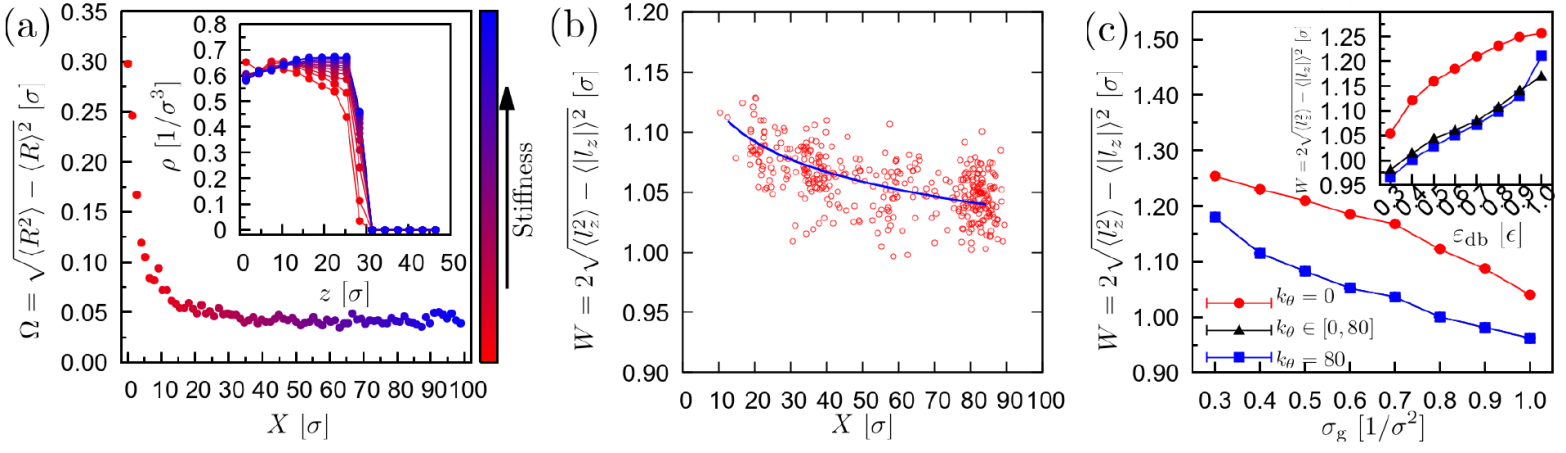
(a) The standard deviation in the end-to-end distance of the brush polymer chains as a function of their grafting position *X*. Larger values of *X* correspond to the stiffer parts of the substrate. (inset) The density profile in the *z* direction at different positions *X*. The color reflects the stiffness of the chains (*k*_θ_). ε_db_ = 0.6 ϵ, σ_g_ = 0.6 σ^–2^. Γ = 0.8 ϵ/rad^2^σ and initial stiffness 0 ϵ/rad^2^ at the softest end. *N* = 4000, *N*_d_ = 10, *N*_b_ = 30 beads. (b) Interpenetration length, *W. l*_*z*_ is the average distance of contact pairs between the droplet and the brush for the same set of parameters as in (a) with *X* here indicating the center-of-mass position of the droplet in the *x* direction along the gradient. (c) Average interpenetration length as a function of the grafting density, σ_g_, for substrates with constant stiffness (*k*_θ_ = 0 ϵ/rad^2^ or *k*_θ_ = 80 ϵ/rad^2^) or with stiffness gradient Γ = 0.8 ϵ/rad^2^σ and initial stiffness 0 ϵ/rad^2^ at the softest end, as indicated. *N* = 4000, *N*_d_ = 10, *N*_b_ = 30 beads. ε_db_ = 0.6 ϵ in the main plot, and σ_g_ = 0.6 σ^–2^ in the inset.

To further understand the durotaxis mechanism of droplets on brush substrates, we have gone one step further and tried to identify the origin of the changing interfacial energy in successful durotaxis cases. In particular, we have measured the standard deviation of the end-to-end distance Ω, which describes the width of the free-end positions distribution of the brush polymer chains (Figure 7a), i.e., the surface roughness of the brush. We can observe that Ω decays monotonically toward the stiffest parts of the brush. In other words, stiffer polymer chains exhibit a smaller extent of fluctuations concerning the end-to-end distance of the polymer chains, which is generally expected when the stiffness increases. Moreover, we can see that the decay of Ω is faster at the soft parts of the substrate and generally follows the decay in the interfacial energy (Figure 6). The inset of Figure 7a presents results for the density profile in the *z* direction at different positions *X* in the *x* direction (along the stiffness gradient). We observe that the thickness of the brush surface becomes smaller toward the stiffer parts, which would correspond to a flatter surface locally. In contrast, slower decaying density profiles correspond to a larger thickness of the brush surface, in other words, to a rougher brush surface, which is observed in the soft substrate parts. In practice, rough surfaces result in a smaller number of contacts with the droplet and, as a result, a higher (less negative) interfacial energy.^13^ On the contrary, a flat profile would allow for a larger number of contacts between the droplet and the substrate. For this reason, the droplet moves toward the stiffer parts of the substrate. We provide further evidence for our argument by measuring the interpenetration length *W* as a function of the position *X* of the center-of-mass of the droplet along the substrate in the direction of the stiffness gradient (Figure 7b). This property reflects the average distance of the substrate–droplet contact pairs, which is noted here with the symbol *l*_*z*_. We can clearly see that the interpenetration length decreases at the stiffer parts of the substrate, which points to a sharper (flatter) brush surface, in accordance with the results of Figure 7a. We have further explored the dependence of *W* on ε_db_ and σ_g_ and found that it decreases as a function of σ_g_ due to the induced stiffness by the steric interactions between the brush polymer chains, while it increases with ε_db_. An important conclusion from the results of Figure 7c is that *W* is clearly larger in the case of soft brushes (*k*_θ_ = 0 ϵ/rad^2^). However, it is the gradual change of this roughness that plays an important role in inducing the interfacial-energy gradient, which in turn translates into the effective force that drives the droplet motion.

**Figure 8:**
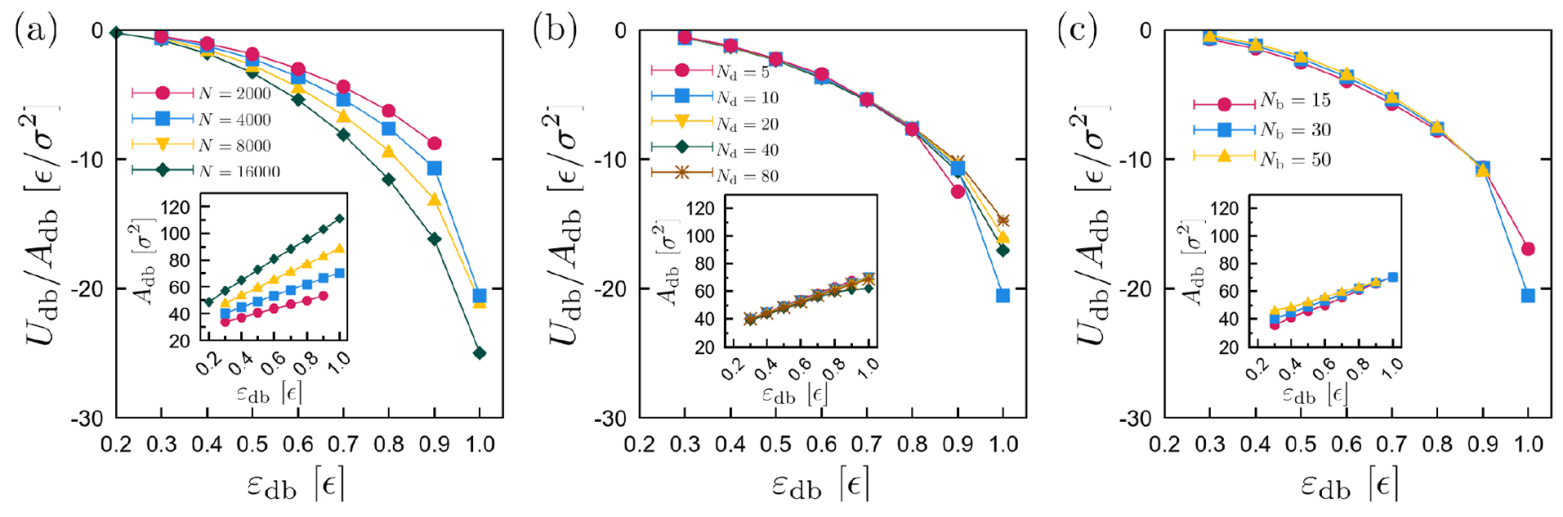
Droplet–Brush interfacial energy *U*_db_ per area of the droplet–substrate contact surface *A*_db_, as a function of ε_db_. σ_g_ = 0.6 σ^–2^ and Γ = 0.8 ϵ/rad^2^σ. Panels show results for different (a) *N* (*N*_b_ = 30 and *N*_d_ = 10 beads), (b) *N*_d_ (*N*_b_ = 30 beads), and (c) *N*_b_ (*N*_d_ = 10 beads). The area *A*_db_ was calculated by using the qhull library,^64^ taking the area of the convex hull.

**Figure 9:**
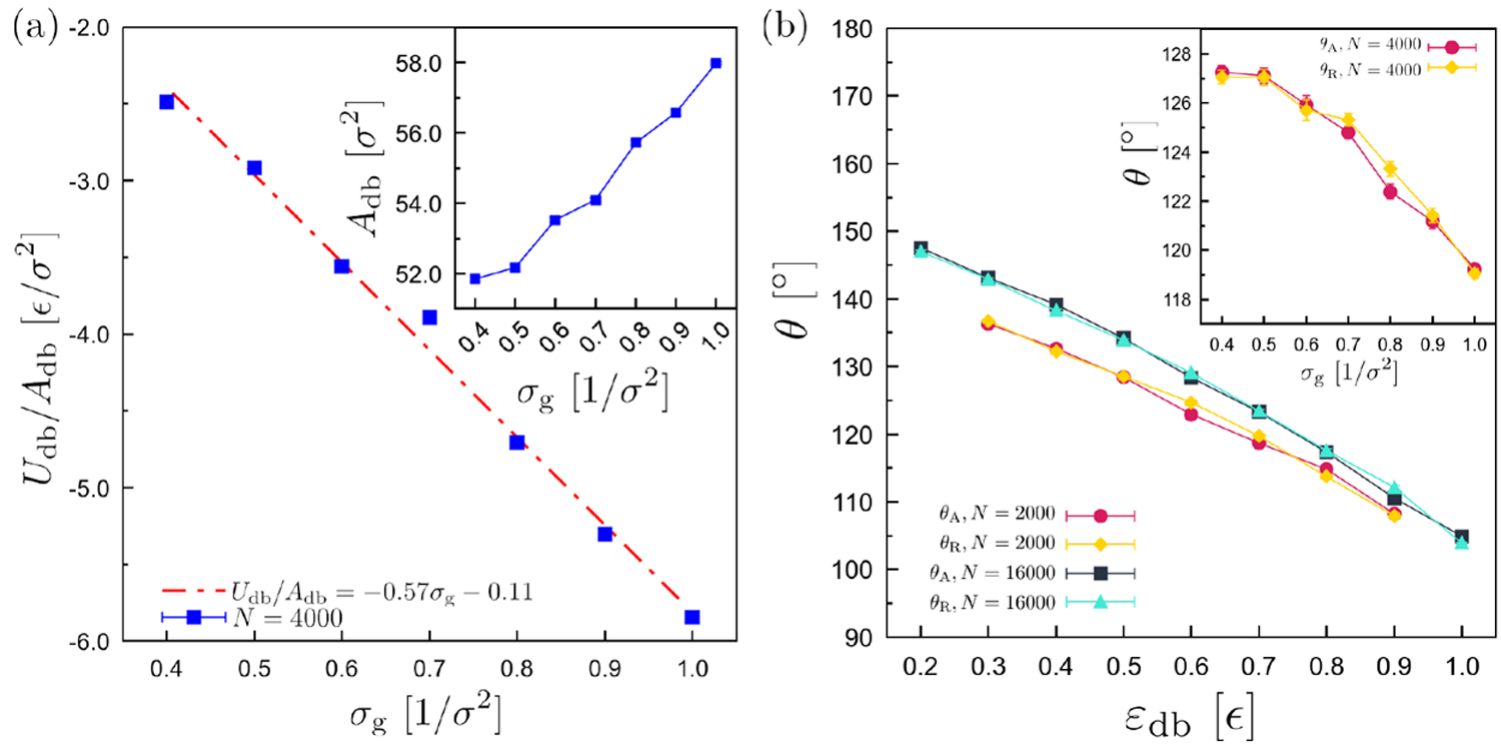
(a) Droplet–Brush average interfacial energy *U*_db_ per area *A*_db_, as a function of the grafting density σ_g_. Dashed-dotted line is the result of a linear fit with slope −0.57 ϵ as indicated. (inset) Illustration of the values of the area *A*_db_ vs the grafting density. ε_db_ = 0.6 ϵ, *N* = 4000, *N*_b_ = 30, and *N*_d_ = 10 beads. Γ = 0.8 ϵ/rad^2^σ. (b) Advancing (θ_A_) and receding (θ_R_) contact angles along the durotaxis motion (*x* direction) as a function of ε_db_ for droplets of different size as indicated. σ_g_ = 0.6 σ^–2^. (inset) The dependence of the contact angles on the grafting density σ_g_ for ε_db_ = 0.6 ϵ. In both the main panel and the inset, *N*_d_ = 10 and *N*_b_ = 30 beads, and Γ = 0.8 ϵ/rad^2^σ.

With these indications that the minimization of the interfacial energy drives the droplet toward areas of more negative energy, in Figure 8, we show the interfacial energy for the same systems shown in Figure 5. When the chain length of the droplet polymer chains *N*_d_ varies within the range considered in our study, there are no noticeable changes in the interfacial energy (Figure 8b), and systems are rather characterized by an equivalent average energy profile, which is reflected in the average velocity of the droplets (Figure 5b). There, we have found that a change in the viscosity of the droplet does not significantly affect the durotaxis efficiency. Slight changes in the average interfacial energy are observed when the brush chain length *N*_b_ varies (Figure 8c). These results are also consistent with those of Figure 5c. In particular, we can see that the brush with chain length *N*_b_ = 50 beads appears to have the lowest average interfacial energy and the largest interfacial area for the whole range of ε_db_ considered here. Moreover, differences in the interfacial energy between the systems with different *N*_b_ become smaller as ε_db_ increases, which is much clearer in the data concerning the interfacial area *A*_db_, in line with the results of Figure 5c. In contrast, rather larger differences are observed when the size of the droplets *N* changes (Figure 8a). In particular, droplets of smaller size have a markedly lower (less negative) interfacial energy per area than larger droplets. These differences become more apparent as the strength of interactions ε_db_ increases. Interestingly, although the energy here shows a monotonic behavior, this is not the case for the average velocity of the droplet (Figure 5a), which indicates that droplets of different size are differently affected in a global sense for a given set of substrate parameters. One contributing factor is that the change in interfacial energy from soft to hard substrate is proportional to the contact area ∝*N*^2/3^, while the mass is proportional to *N*. Therefore, the energy change per atom is ∝*N*^–1/3^, other factors being equal, and the velocity change can be expected to scale as *N*^–1/6^. This is not inconsistent with Figure 5a at medium strength ε_db_. Finally, the interfacial energy decreases proportionally to the grafting density, with a slope of −0.57 ϵ (Figure 9a), suggesting a proportionally larger number of contacts between the droplet and the substrate as the grafting density grows.

We have also monitored the advancing (θ_A_) and receding (θ_R_) contact angles during the durotaxial motion, and results are presented in Figure 9b for typical cases. In this case, the angles have been determined by using the curvature of the droplet as described in previous studies,^65^ thus avoiding error-prone fits. We observe that both θ_A_ and θ_R_ decrease rather linearly with the increase of ε_db_. A linear decrease has been observed for droplets on solid substrates.^13,65^ Droplets of different size, namely, *N* = 2000 and *N* = 16 000 beads, show the same trend. Moreover, as ε_db_ increases, the differences between smaller and larger droplets become less pronounced. A much weaker dependence of the contact angles on the grafting density has been observed for σ_g_ ≤ 0.6 σ^–2^ and a small scale rather linear dependence for σ_g_ ≥ 0.6 σ^–2^. Our results, which are averaged over the whole trajectory, do not show any statistically significant difference between advancing and receding contact angles. Due to the greater magnitude of interfacial energy (more negative) at the stiffer parts of the substrate (Figure 6) one would expect a smaller advancing contact angle (e.g., as Figure 9b suggests, a larger attraction leads to smaller contact angles). Slightly larger values for the receding contact angle θ_R_ are observed consistently for different values of σ_g_ and ε_db_ but, still, within the statistical error. In experiment and theory, when the droplet moves by steadily applied external force the receding contact angles are smaller than the advancing ones in which case friction effects might also play a role.

**Figure 10:**
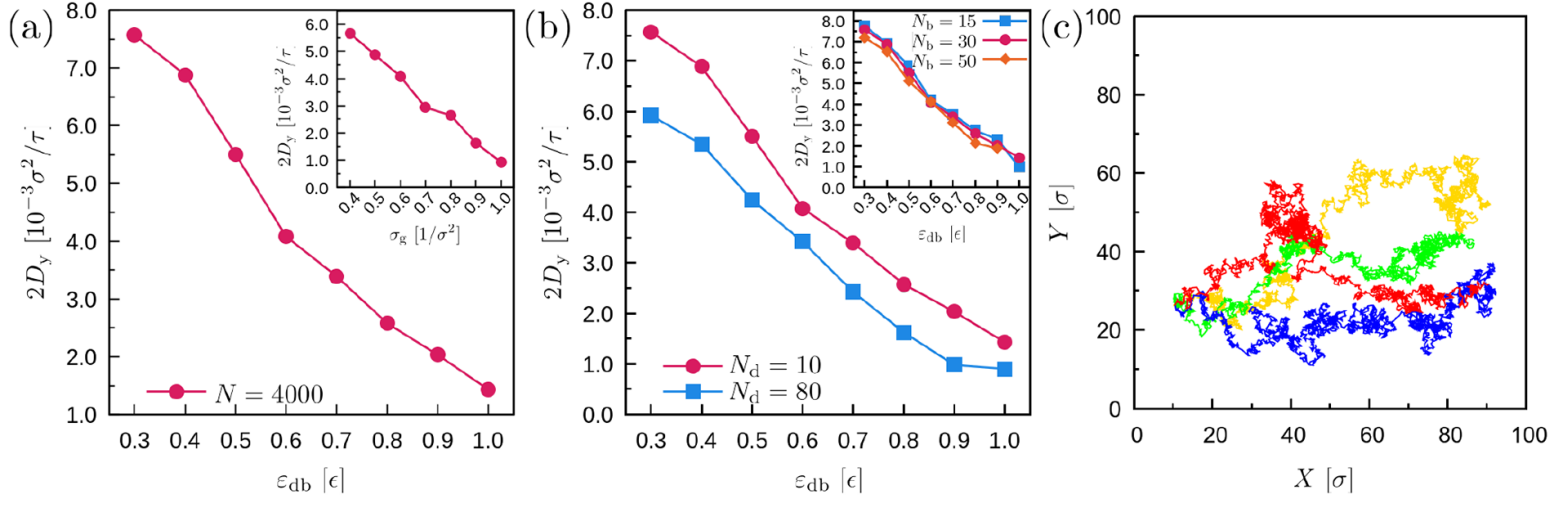
Diffusion coefficient (σ_g_ = 0.6 σ^–2^) as a function of (a) ε_db_. (inset) The dependence on the grafting density σ_g_ for the case ε_db_ = 0.6 ϵ. *N*_d_ = 10 and *N*_b_ = 30 beads. (b) Diffusion coefficient for different droplet chain length *N*_d_ and brush chain length *N*_b_ (inset) as a function of ε_db_. σ_g_ = 0.6 σ^–2^, ε_db_ = 0.6 ϵ in the inset. *N* = 4000 beads. (c) Typical, successful durotaxis trajectories of the center of mass of the droplet in the *x*–*y* space as indicated by different colors for a separate run. σ_g_ = 0.6 σ^–2^, ε_db_ = 0.6 ϵ, *N* = 4000, *N*_b_ = 30, and *N*_d_ = 10 beads, and Γ = 0.8 ϵ/rad^2^σ.

Finally, to investigate the diffusion of the droplet and how it is affected by the main parameters, we have analyzed the movement of the center of mass of the droplet in the *y* direction (Figure 10). Dynamics in the *y* direction is free of extraneous effects due to grafting density gradient and the durotaxis itself, so it serves as a good control and measurement of the diffusion properties. Overall, droplets with higher viscosity and larger wettability on the brush substrate show a slower diffusion. The same effect has the grafting density. In contrast, *N*_b_ does not have a tangible effect on the droplet motion. In Figure 10c, we present typical trajectories as a function of the *X* and *Y* positions of the droplet center-of-mass. We can observe that durotaxis motion is also characterized by significant random motion in both *x* and *y* axes including “reversals”. In some cases, we can observe that the droplet can cover in the *y* direction a distance as much as 30% of the total distance in the *x* direction, which suggests that durotaxis motion is also strongly affected by random effects at the interface between the droplet and the substrate, especially when the gradient of the interfacial energy becomes small; i.e., the driving force of the durotaxis is correspondingly small.

## Conclusions

In this study, we have proposed a new design of brush polymer substrates that is capable of leading to the durotaxis motion of nanodroplets. The knowledge gained here may lead to new experimental brush-based substrate designs and provide further understanding of relevant biological processes, such as the motion of cells on tissues^11,12^ or the mucus flow around lung cilia.^55^ Our analysis has also indicated that the durotaxis motion on brush substrates is driven by a corresponding gradient in the interfacial energy between the droplet and the substrate, in line with previous findings in the context of various other substrate designs.^13,16,24^ Moreover, we have found that the origin of the steady increase of the interfacial energy is related to the state of the brush surface, which appears as a “rougher” profile in the softer parts of the substrate and a flatter interface in the stiffer regions. This translates into a larger number of contacts between the droplet and the substrate in the stiffer parts and, hence, a more negative interfacial energy along the direction of the stiffness gradient.

We have also conducted a parametric study based on the various system parameters in order to gain further insight into the system and identify the key parameters of the brush substrate design. Two of the key parameters are the grafting density of the polymer chains and the substrate wettability. Our findings suggest that the durotaxis motion is favored by a moderate grafting density, which in our case translates to values of grafting density σ_g_ ≈ 0.5 σ^–2^ and at the same time moderate values of the substrate wettability, namely, 0.5 ϵ ≤ ε_db_ ≤ 0.9 ϵ. Surprisingly, we have found that the stiffness gradient itself, as defined by the linear change in the stiffness of individual polymer chains tuned by the harmonic constant *k*_θ_, does not induce important changes in the efficiency of the durotaxis motion. This might be due to the relatively large grafting density, which affects the apparent stiffness of the polymer chains, thus minimizing the actual effect of the gradient. Such an effect has not been observed in previous systems.^13^ Moreover, the droplet viscosity also seems not to affect the durotaxis efficiency as also the length of the polymer brush chains, since the durotaxis performance seems to saturate after a certain polymer length. In contrast, the size of the droplet plays a role. In particular, smaller droplets seem to reach a faster durotaxis and a lower adhesion to the subtrate, while larger droplets move with a lower average velocity exhibiting their maximum velocity at larger droplet–substrate adhesion. Furhtermore, we have not identified any tangible differences between the advancing and receding contact angles during the durotaxis motion. The durotaxis motion is induced by tiny effects at the droplet–substrate interface as judged by the gradient in the droplet–substrate interfacial energy. Hence, there is a back-and-forth wiggling motion while the brush slowly guides the droplet toward a lower-energy state. Finally, we have discussed how our findings could motivate further experimental research in the area of self-sustained fluid motion on brush gradient substrates. It would also be interesting to investigate the droplet durotaxis behavior when many droplets are placed onto the substrate and explore various effects, such as droplet coalescence or how the droplet–substrate interactions are affected in populations of droplets. Further work to explore such possibilities is expected in this direction in the future. We anticipate that this study for the first time presents new possibilities for the implementation and understanding of durotaxis motion of fluids on brush substrates with important implications for various areas, for example, in the context of biology.

## Supporting Information Available

Movie illustrates an example of durotaxis motion onto a gradient brush substrate. The parameters for the system are *N* = 4000, *N*_d_ = 10, and *N*_b_ = 30 beads. σ_g_ = 0.6 σ^–2^, Γ = 0.8 ϵ/rad^2^ σ (chains at the very soft right end are fully flexible, i.e., *k*_θ_ = 0 ϵ/rad^2^, while at the very stiff part *k*_θ_ = 80 ϵ/rad^2^), and ε_db_ = 0.6 ϵ (MP4)
